# Supplementary material for: Physiography, foraging mobility, and the first peopling of Sahul
Source: Nat Commun. 2024 Apr 23;15:3430. doi: 10.1038/s41467-024-47662-1 (PMC11039755; doi:10.1038/s41467-024-47662-1)
Supplement: Supplementary file 1 — Supplementary Information [file 41467_2024_47662_MOESM1_ESM.pdf]

SUPPLEMENTARY INFORMATION

Physiography, foraging mobility, and the first peopling of Sahul

Tristan Salles[1*], Renaud Joannes-Boyau[2], Ian Moffat[2,3], Laurent Husson[4] and Manon Lorcery[1,4]

[1]School of Geosciences, The University of Sydney, Sydney, Australia
[2]Geoarchaeology and Archaeometry Research Group, Southern Cross University, Lismore, Australia
[3]Archaeology, College of Humanities, Arts and Social Sciences, Flinders University, Adelaide, Australia
[4]ISTerre, CNRS, Université Grenoble-Alpes, Grenoble, France

**corresponding authors: TS: tristan.salles@sydney.edu.au*

INDEX

Supplementary Figure 1. Predicted landscape evolution at 35 ka.

Supplementary Figure 2. Physiographic variables used to evaluate resistance to movement at 65 ka.

Supplementary Figure 3. Examples of physiographic resistance to movement for the entire Sahul landmass.

Supplementary Figure 4. Examples of migration pathways from two mechanistic simulations with different entry points.

Supplementary Figure 5. Evaluation of human migration and likelihood of occupation from mechanistic movement simulation.

Supplementary Figure 6. Probability of Lévy walkers' presence over modern-day Australia.

Supplementary Figure 7. Regional morphological changes around identified sites with predicted archaeological potential.

Supplementary Table 1. List of archaeological sites.

Supplementary Table 2. Hierarchical classification values.

Supplementary Table 3. Archaeological locations (coordinates and names).

Supplementary Table 4. Coordinates and regional morphological context of predicted sites.

Supplementary References

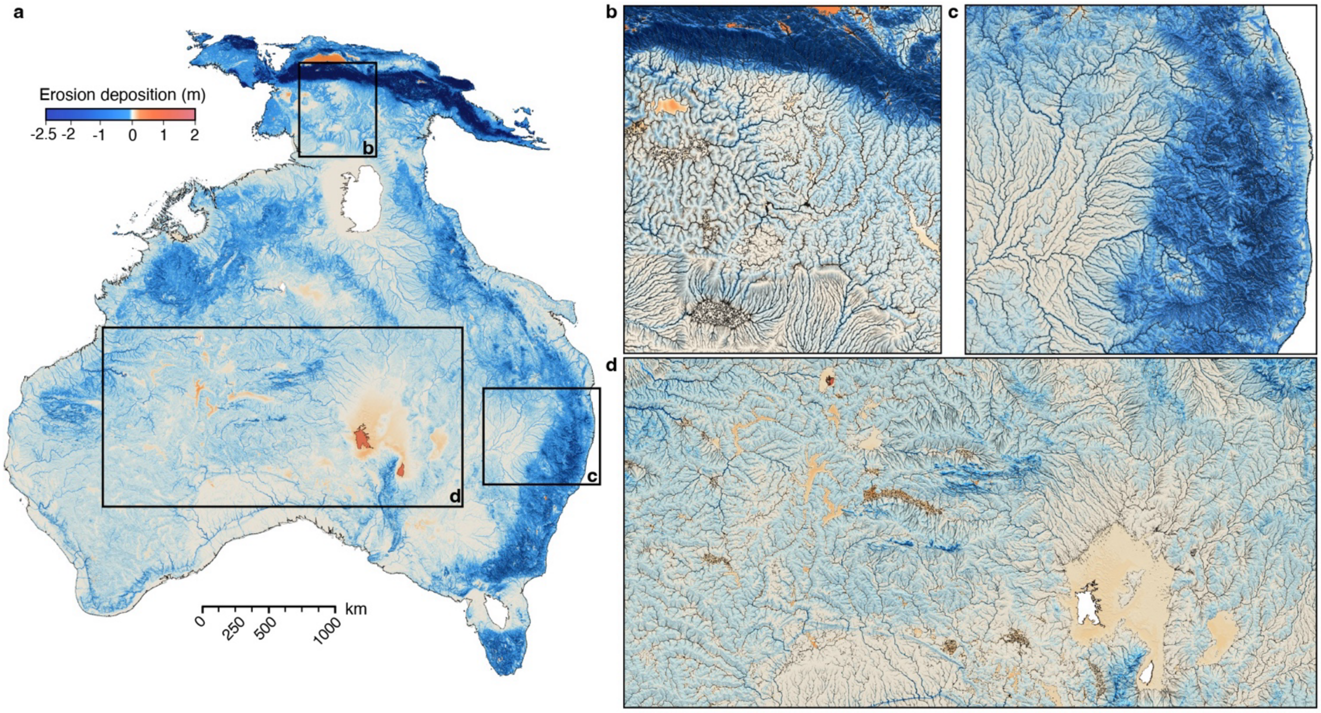

Supplementary Figure 1. Predicted landscape evolution at 35 ka. a. Simulated cumulative erosion (blue) and deposition (red) in meters induced by riverine and hillslope processes, highlighting differential erosion across mountain ranges (e.g., New Guinea & Great Australian Escarpment) and sediment accumulation in endorheic basins, lakes, and alluvial plains. Zooms over selected regions (**b**, **c**, and **d**) defined in a and overlaid with drainage patterns from main river systems predicted by the multiple flow direction algorithm. Panels show the results from goSPL software and are visualised with the open-source Paraview software (https://www.paraview.org).

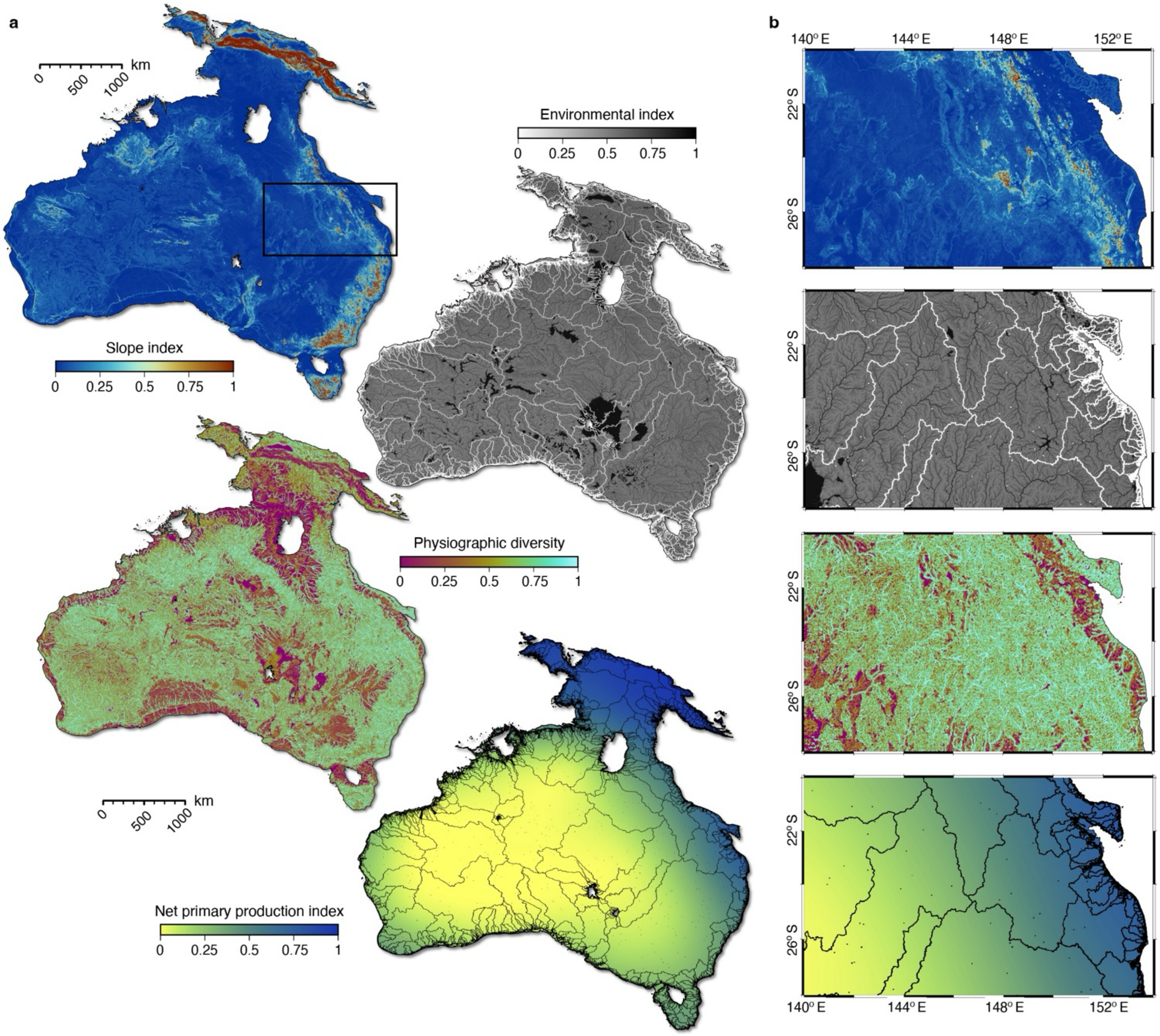

Supplementary Figure 2. Physiographic variables used to evaluate resistance to movement at 65 ka. a. Normalized Sahul relief based on mean slopes for the continental region computed over the 2 km grid (top panel). The environmental index (second panel) accounts for lakes, rivers, and arid regions (e.g., central Australia). Values close to 1 will act as barriers and impede movement across the region. The river-related component of the index varies with the simulated water flux. Overlaid white lines represent predicted main drainage divides. Third panel shows the physiographic diversity index from which the landscape complexity (Shannon index) is derived. Bottom panel presents the net primary production index at 65 ka based on LOVECLIM Earth system model[1,2]. **b.** Local distribution of the above variables from a region in Eastern Australia (black rectangle – defined in the top panel of **a**). All data are available under Creative Commons Attribution 4.0 International Licence (http://creativecommons.org/licenses/). The open-source python interface for the Generic Mapping Tools (https://www.pygmt.org) is used for visualization.

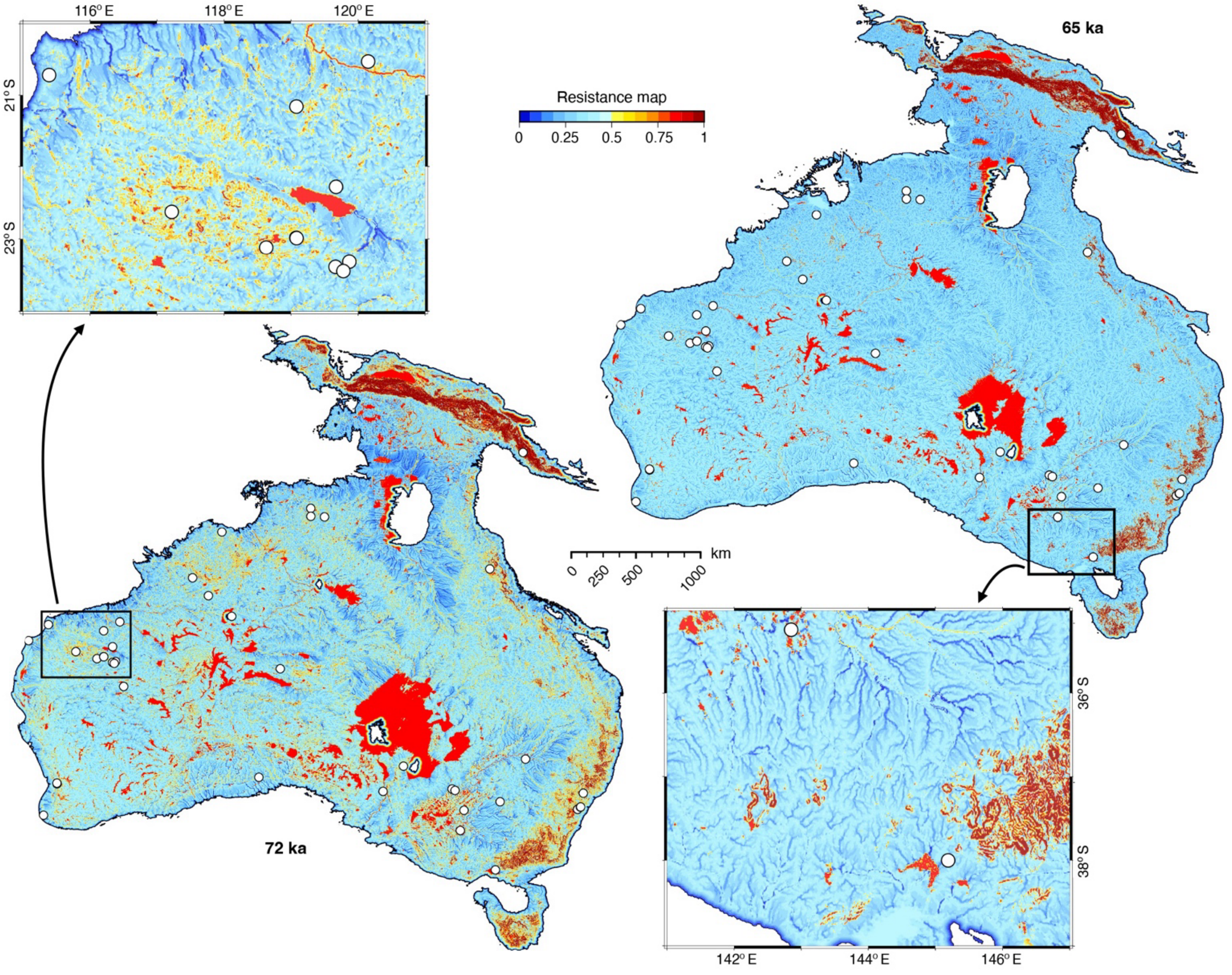

Supplementary Figure 3. Examples of physiographic resistance to movement for the entire Sahul landmass. Normalized cost map on the left corresponds to the driest period at ~72 ka, with a coastline at -49 m isobath[3]. The one at ~65 ka, on the right, combines a wetter period with a low sea-level position (coastline at -85 m isobath[3]). These maps are defined from topographic slopes, environmental factors (rivers, lakes, and deserts), net primary production, and landscape complexity. The cost quantifies the local resistance of the landscape to displacement (cost values increase with resistance). Low cost areas are also added along coastal regions up to 50 km inland. The positions of the archaeological sites older than 35 ka[4] are represented with white circles. Focus on two regions located in the Pilbara (top left) and in the southern Murray-Darling Basin (bottom right). The open-source python interface for the Generic Mapping Tools (https://www.pygmt.org) is used for visualization.

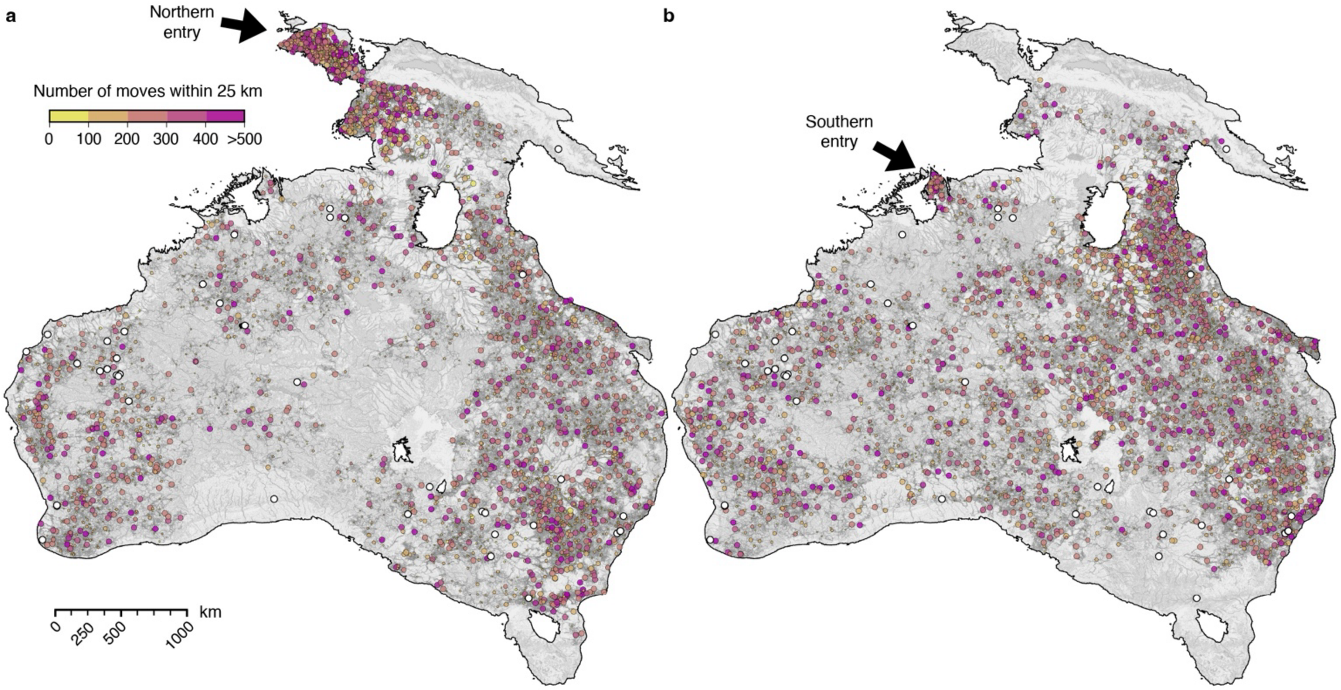

Supplementary Figure 4. Examples of migration pathways from two mechanistic simulations with different entry points. Lévy walk simulations of human migration across Sahul using the mechanistic spatially explicit simulation SiMRiv[5]. Two scenarios are presented, one with an entry point in northwest Papua from the Bird's Head Peninsula[6,7,8] (**a**) and the other one with an entry point in the Kimberley region of Western Australia[9] (**b**). Both simulations predict migrations routes passing through 39 of the 40 archaeological sites (Supplementary Tab. 1). Colours represent the number of walker moves between consecutive circles and the size of the circle is scaled based on the cumulative distance travelled by walkers for each 25 km segment. Generated paths using the SimRiv software and visualised with the open-source python interface for the Generic Mapping Tools (https://www.pygmt.org).

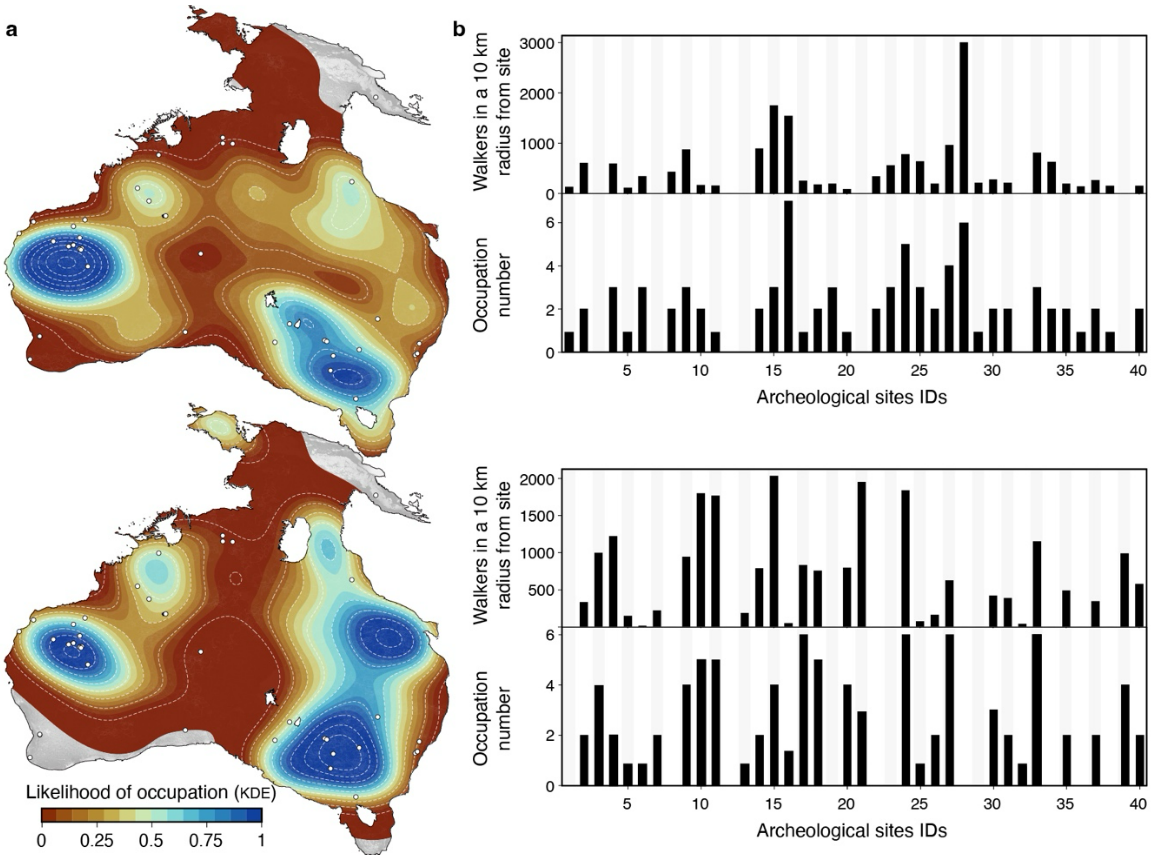

Supplementary Figure 5. Evaluation of human migration and likelihood of occupation from mechanistic movement simulation. a. Statistical analysis of peopling of Sahul using kernel density estimation based on simulated pathways for two realisations using the northern entry point. **b.** The number of walkers reaching archaeological sites within a 10 km radius (Supplementary Tab. 1) is presented on top panels for each simulation. In the bottom panels, distinct visits to considered sites (e.g., occupation number) are obtained from clustering based on cumulative travelled distance. For the 40 archaeological sites, the sampling size n is obtained from the 10 million walker steps aggregated assuming a 10 km radius of knowledge. Maps are produced with the open-source python interface for the Generic Mapping Tools (https://www.pygmt.org) based on paths generated with SimRiv software.

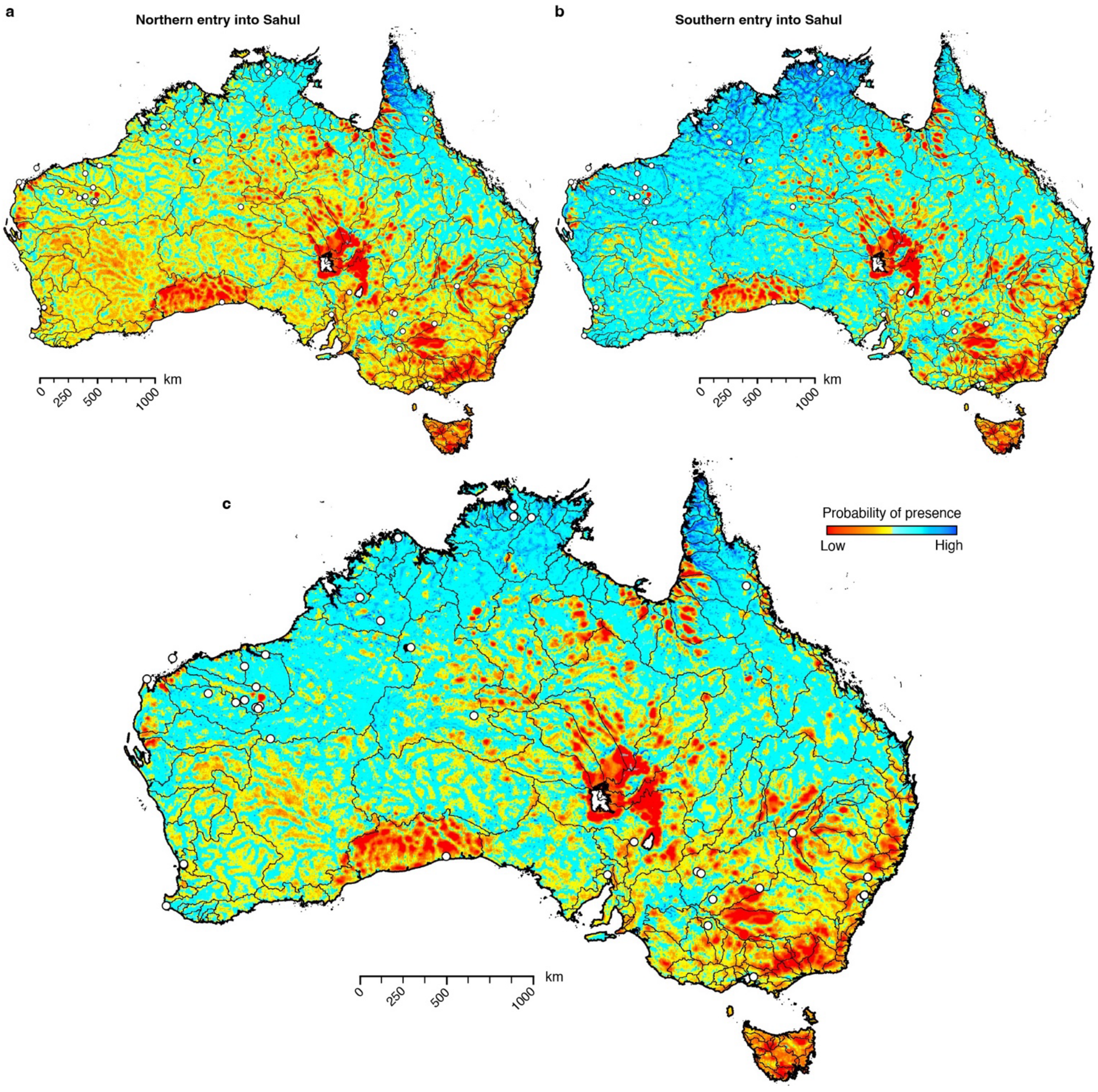

Supplementary Figure 6. Probability of Lévy walkers' presence over modern-day Australia. Heat map of aggregated walkers' number into 0.05° cells clipped to Australia considering the northwest Papua entry point[6,7,8] (**a**), the Kimberley region[9] (Western Australia – **b**, southern entry) and when combining both routes (**c**). Cyan to blue areas correspond to regions with high probability of presence and red to yellow areas are regions which are never or rarely chosen. White circles indicate locations of archaeological sites (Supplementary Tab. 1). Maps are produced with the open-source python interface for the Generic Mapping Tools (https://www.pygmt.org) based on paths generated with SimRiv software.

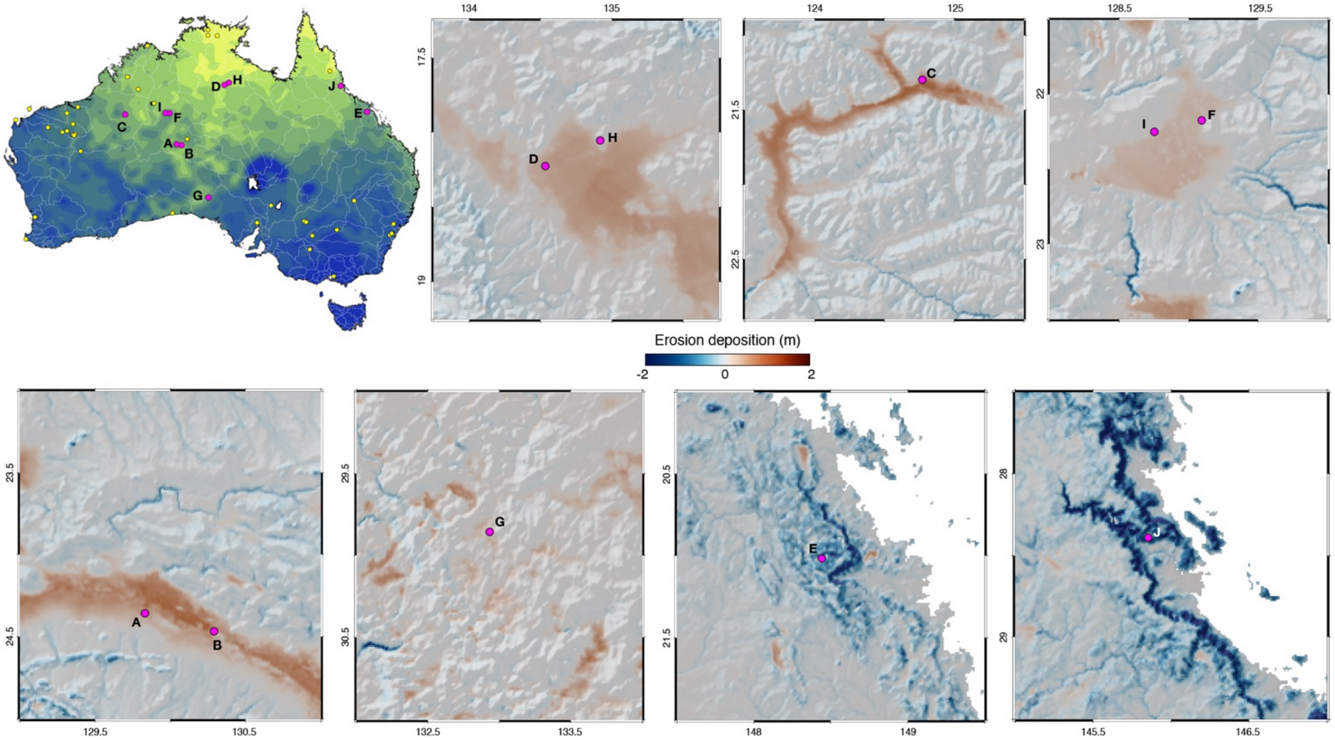

Supplementary Figure 7. Regional morphological changes around identified sites with predicted archaeological potential. Results from the landscape evolution model showing cumulative erosion deposition on 2×2° tiles for sites A to J (Supplementary Tab. 4) based on the mechanistic movement realisations (Fig. 7). Top left map is produced with the open-source python interface for the Generic Mapping Tools (https://www.pygmt.org) and other panels are the results from goSPL software and are visualised with the open-source Paraview software (https://www.paraview.org).

Longitude	Latitude	Site Name	Site ID	Reference
129.42	-31.48	Allen's Cave	1	Roberts et al. 1996
145.19	-38	Bend Road	2	Hewitt & Allen 2010
115.39	-20.72	Boodie Cave	3	Veth et al. 2017
142.85	-35.23	Box Gully (Lake Tyrell)	4	Richards et al. 2007
125	-17.43	Carpenter's Gap 1	5	O'Connor 1995; Langley et al. 2016; Maloney et al. 2018; McConnell & O'Connor 1997; Wallis 2001
150.67	-33.73	Cranebrook Terrace	6	Nanson et al. 1987
147.2	-30.2	Cuddie Springs	7	Gillespie & Brook 2006; Grün et al. 2010
115.07	-34.15	Devils Lair	8	Dortch 1979a, 1979b; Dortch & Dortch 1996; Turney et al. 2001
118.63	-23.12	Djadjiling	9	Morse 2009; Law et al. 2010
119.0777	-22.9845	HD07-3A-PAD13	10	Cropper 2018a
119.083	-22.9897	HS-A1	11	Cropper 2018b
147.03	-8.56	Ivane Valley	12	Summerhayes et al. 2010
114.07	-21.85	Jansz	13	Przywolnik 2002
117.2196	-22.6276	Juukan 2	14	Reynen 2018
119.671636	-22.28119	Kakutungutanta (CB10-93)	15	Dias and Rapley 2014
120.41	-25.08	Karnatukul (Serpent's Glen Rockshelter)	16	McDonald et al. 2018
142.3	-32.3	Lake Menindee	17	Cupper and Duncan 2006
142.5	-32.4	Lake Tandou	18	Balme and Hope 1990
132.88	-12.5	Madjedbebe (Malakunanja II)	19	Clarkson et al. 2017; Roberts et al. 1990; Roberts & Jones 1994
126.96	-14.2	Minjiwarra	20	Veth et al. 2019
145.5	-33.2	Nombinnie	21	Bowler et al. 2012
132.89	-13.06	Nauwalabila 1	22	Roberts et al. 1990, 1993
133.8	-13.1	Nawarla Gabarnmang	23	David et al. 2011, 2013, 2019
119.87	-23.32	Newman Rock Shelter	24	Slack et al. 2018
144.8158	-16.8034	Ngarrabullgan Cave	25	Bird et al. 1999; David 1993
137.7024	-32.4751	PACD H1	26	Walshe 2012
119.66348	-23.3908	PAD10-17	27	Slack et al. 2018
127.61	-20.14	Parnkupirti	28	Veth et al. 2009
115.967249	-31.93851	Perth Airport: Adelaide Street	29	Dortch & Dortch 2019
150.8569	-33.583	PT 12-A(2)	30	Williams et al. 2014
150.8718	-33.57	PT 12-B	31	Williams et al. 2014
130.85	-23.83	Puritjarra	32	Smith 2010
119.78	-23.45	RH12-01	33	Slack et al. 2018
126.06	-18.69	Riwi	34	Balme 2000; Wood et al. 2016
151.05	-32.6	Sandy Hollow	35	Hughes et al. 2014
115.96	-31.89	Upper Swan	36	Pearce & Barbetti 1981
139.06	-30.69	Warratyi	37	Hamm et al. 2016
120.15	-20.53	Watura Jurnti	38	Marsh et al. 2018
143.1	-33.8	Willandra Lakes/Mungo	39	Barbetti 1973; Bell 1991; Bowler et al. 2012; Bowler et al. 2003; Bowler and Price 1998; Clark 1987; Huxtable and Aitken 1977; Johnston & Clark 1998; McBryde 1980; Olley et al. 2006; Oyston 1996; Thorne et al. 1999
119.08	-21.16	Yurlu Kankala	40	Morse et al. 2014; Reynen et al. 2018

Supplementary Table 1. List of archaeological locations (coordinates and names) with their corresponding references from Crabtree *et al.*[4] for 40 sites distributed across Sahul landmass with ages ≥ 35,000 years.

	**Category**						
	**1**	**2**	**3**	**4**	**5**	**6**	**7**
**Slope** (degrees)	<0.125	≥0.125	≥0.25	≥0.5	≥0.75	≥1.5	≥3
**Water flux** (Log-scale m^3/yr)	<7	≥7	≥8	≥9	≥10		

Coarse scale TPI_{SC}

**Fine scale** TPI_{SF}	**Coarse scale** TPI_{SC} < -100	-100 to 100	> 100
> 100	**3**<br>Upland incised drainages Stream headwaters	**7**<br>Flat ridge tops Mesa tops	**10**<br>Mountain tops High narrow ridges
-100 to 100	**2**<br>Midslope incised drainages Local valleys in plains	**5** or **6**<br>**5** – Broad flat areas (slope<0.125)<br>**6** – Broad open slopes (slope≥0.125)	**9**<br>Midslope drainage divides Local ridges in plains
< -100	**1**<br>V-shape river valleys Deep canyons	**4**<br>U-shape valleys	**8**<br>Local ridge/hilltops within broad valleys

Supplementary Table 2. Hierarchical classification values. Top table shows the parametrization for the slope and water flux variables. Bottom table describe the classification of landforms[10] made by combining the two observation scales of the standardized topographic position index ($TPI_{SF}|TPI_{SC}$ for fine and coarse respectively).

Longitude	Latitude	Name	Mean age | max | min	Nb ages	Rating	Reference
129.42	-31.48	Allen’s Cave	39800 | 39800 | 39800	1	A	Roberts et al. 1996
145.19	-38	Bend Road	35300 | 35300 | 35300	1	A	Hewitt & Allen 2010
115.39	-20.72	Boodie Cave	45357 | 53900 | 35294	8	A	Veth et al. 2017
142.85	-35.23	Box Gully (Lake Tyrell)	40384 | 40384 | 40384	1	B	Richards et al. 2007
125	-17.43	Carpenter’s Gap 1	41156 | 43357 | 39220	10	A	O’Connor 1995; McConnell & O’Connor 1997; Wallis 2001; Langley et al. 2016; Maloney et al. 2018
150.67	-33.73	Cranebrook Terrace	40323 | 47000 | 36700	11	B/C	Nanson et al. 1987
147.2	-30.2	Cuddie Springs	42470 | 51000 | 35400	12	B	Gillespie & Brook 2006; Grun et al. 2010
115.07	-34.15	Devils Lair	42557 | 48130 | 35160	14	A/C	Dortch 1979 / 1979a; Dortch & Dortch 1996; Turney et al. 2001
118.63	-23.12	Djadjiling	35436 | 35753 | 35159	3	A	Morse 2009; Law et al. 2010
119.0777	-22.9845	HD07-3A-PAD13	41550 | 47100 | 36000	2	A	Cropper 2018:181
119.083	-22.9897	HS-A1	35396 | 35617 | 35175	2	A	Cropper 2018:220
147.03	-8.56	Ivane Valley	39360 | 41951 | 35049	7	A	Summerhayes et al. 2010
114.07	-21.85	Jansz	35230 | 35230 | 35230	1	A	Przywolnik 2002
117.2196	-22.6276	Juukan 2	39246 | 41100 | 38020	3	A/B	Reynen 2018:153
119.671636	-22.28119	Kakutungutanta (CB10-93)	36039 | 36039 | 36039	1	A	Dias and Rapley 2014
120.41	-25.08	Karnatukul (Serpent’s Glen Rockshelter)	44104 | 47039 | 39345	3	A	McDonald et al. 2018
142.3	-32.3	Lake Menindee	42315 | 43100 | 41530	2	A	Cupper and Duncan 2006
142.5	-32.4	Lake Tandou	36000 | 36000 | 36000	1	B	Balme and Hope 1990
132.88	-12.5	Madjedbebe (Malakunanja II)	53729 | 64900 | 36500	21	A	Roberts et al. 1990; Roberts & Jones 1994; Clarkson et al. 2017
126.96	-14.2	Minjiwarra	43550 | 49300 | 37800	2	A	Veth et al. 2020
145.5	-33.2	Mungo	37913 | 41740 | 35900	3	B	Bowler et al. 2012
132.89	-13.06	Nauwalabila 1	53400 | 53400 | 53400	1	A	Roberts et al. 1990 / 1993
133.8	-13.1	Nawarla Gabarnmang	41183 | 45072 | 35091	36	A	David et al. 2019
119.87	-23.32	Newman Rock Shelter	35250 | 35250 | 35250	1	A	Slack et al. 2018
144.8158	-16.8034	Ngarrabullgan Cave	35386 | 35500 | 35200	3	A	Bird et al. 1999
137.7024	-32.4751	PACD H1	41599 | 48100 | 36600	5	A/B/C	Walshe 2012
119.66348	-23.3908	PAD10-17	35450 | 35450 | 35450	1	A	Slack et al. 2018
127.61	-20.14	Parnkupirti	44866 | 52400 | 37200	3	A	Veth et al. 2009
115.967249	-31.93851	Perth Airport: Adelaide Street	35000 | 35000 | 35000	1	A	Dortch & Dortch 2019
150.8569	-33.583	PT 12-A(2)	36000 | 36000 | 36000	1	A	Williams et al. 2013
150.8718	-33.57	PT 12-B	36000 | 36000 | 36000	1	A	Williams et al. 2013
130.85	-23.83	Puritjarra	41966 | 46000 | 35100	3	B/C	Smith et al. 1997; Smith 2010
119.78	-23.45	RH12-01	42020 | 42020 | 42020	1	A	Slack et al. 2018
126.06	-18.69	Riwi	42023 | 48800 | 35300	37	A	Wood et al. 2016
151.05	-32.6	Sandy Hollow	52900 | 52900 | 52900	1	B	Hughes et al. 2014
115.96	-31.89	Upper Swan	37233 | 39500 | 35100	3	A	Pearce & Barbetti 1981
139.06	-30.69	Warratyi	41352 | 44400 | 35260	5	A	Hamm et al. 2016
120.15	-20.53	Watura Jurnti	39217 | 39677 | 38772	3	A	Marsh et al. 2018
143.1	-33.8	Willandra Lakes	43509 | 59000 | 35300	42	A	Bowler and Price 1998; Bowler et al. 2003; Huxtable and Aitken 1977; Oyston 1996; Olley et al. 2006
119.08	-21.16	Yurlu Kankala	40339 | 40752 | 39819	4	A	Morse et al. 2014; Reynen et al. 2018

Supplementary Table 3. Archaeological locations (coordinates and names) as in Supplementary Tab. 1 with their corresponding age range, quality rating and sources from Crabtree *et al.*[4].

Longitude	Latitude	Site ID	Regional context
130.30	-24.46	A	SE of Puritjarra (site 32 in Extended Data Tab. 1) on the edges of Lake Neale (Central Australia)
129.83	-24.36	B	SE of Puritjarra (site 32 in Extended Data Tab. 1) on the edges of Lake Neale (Central Australia)
124.78	-21.3	C	Along the banks of the Nullagine River in the Pilbara region (Western Australia)
134.93	-18.05	D	Wetlands on the western side of Tarrabool Lake connecting drainage from Beetaloo Basin and Barkly Tablelands (Northern Territory)
148.45	-21.02	E	At the bottom of the Massey Creek incised valley in the Atherton Tablelands (Queensland)
129.1	-22.17	F	Northern end of Lake Mackay (Western Desert region) on the Northern Territory side of the lake
132.93	-29.86	G	South of Tallaringa Conservation Park on the fringe of the Great Victoria Desert (South Australia)
134.53	-18.22	H	Wetlands on the western side of Tarrabool Lake connecting drainage from Beetaloo Basin and Barkly Tablelands (Northern Territory)
128.75	-22.25	I	Northern end of Lake Mackay (Western Desert region) on the Northern Territory side of the lake
145.85	-18.38	J	Lower Herbert River near Lumholtz in the Atherton Tablelands (Cassowary Coast Region, Queensland)

Supplementary Table 4. Coordinates and regional morphological context of predicted sites with archaeological potentials based on the mechanistic movement realisations (Fig. 7 and Supplementary Fig. 7).

Supplementary References

1. Bradshaw, C. *et al.* Minimum founding populations for the first peopling of Sahul. *Nat. Ecol. & Evol.* **3**, DOI: 10.1038/ s41559-019-0902-6 (2019).
2. Claussen, M. *et al.* Earth system models of intermediate complexity: Closing the gap in the spectrum of climate system models. *Clim. Dyn.* **18**, 579–586, DOI: 10.1007/s00382-001-0200-1 (2002).
3. Lambeck, K. & Chappell, J. Sea level change through the last glacial cycle. *Science* **292**, 679–686, DOI: 10.1126/science. 1059549 (2001).
4. Crabtree, S. *et al.* Landscape rules predict optimal superhighways for the first peopling of Sahul. *Nat. Hum. Behav.* **5**, DOI: 10.1038/s41562-021-01106-8 (2021).
5. Quaglietta, L. & Porto, M. SiMRiv: An R package for mechanistic simulation of individual, spatially-explicit multistate movements in rivers, heterogeneous and homogeneous spaces incorporating landscape bias. *Movement Ecology* DOI:10.1186/s40462-019-0154-8 (2019).
6. Kealy, S., Louys, J. & O'Connor, S. Least-cost pathway models indicate northern human dispersal from Sunda to Sahul. *Journal of Human Evolution* **125**, 59–70, DOI: 10.1016/j.jhevol.2018.10.003 (2018).
7. Kealy, S., Louys, J. & O'Connor, S. Islands under the sea: A review of early modern human dispersal routes and migration hypotheses through wallacea. *The J. Island Coast. Archaeol.* **11**, 364–384, DOI: 10.1080/15564894.2015.1119218 (2016).
8. Bird, M. *et al.* Early human settlement of Sahul was not an accident. *Sci. Reports* **9**, 8220, DOI: 10.1038/ s41598-019-42946-9 (2019).
9. Bradshaw, C. J. *et al.* Directionally supervised cellular automaton for the initial peopling of Sahul. *Quat. Sci. Rev.* **303**, 107971, DOI: 10.1016/j.quascirev.2023.107971 (2023).
10. Weiss, A. Topographic position and landforms analysis. In *ESRI Users Conference, San Diego, CA.* (2001).
