# Supplementary material for: Physiography, foraging mobility, and the first peopling of Sahul
Source: Nat Commun. 2024 Apr 23;15:3430. doi: 10.1038/s41467-024-47662-1 (PMC11039755; doi:10.1038/s41467-024-47662-1)
Supplement: Supplementary file 3 — Reporting Summary [file 41467_2024_47662_MOESM3_ESM.pdf]

nature portfolio

Corresponding author(s): Tristan Salles

Last updated by author(s): Mar 28, 2024

Reporting Summary

Nature Portfolio wishes to improve the reproducibility of the work that we publish. This form provides structure for consistency and transparency in reporting. For further information on Nature Portfolio policies, see our Editorial Policies and the Editorial Policy Checklist.

Statistics

For all statistical analyses, confirm that the following items are present in the figure legend, table legend, main text, or Methods section.

n/a	Confirmed	
☐	☒	The exact sample size (*n*) for each experimental group/condition, given as a discrete number and unit of measurement
☐	☒	A statement on whether measurements were taken from distinct samples or whether the same sample was measured repeatedly
☒	☐	The statistical test(s) used AND whether they are one- or two-sided<br>*Only common tests should be described solely by name; describe more complex techniques in the Methods section.*
☒	☐	A description of all covariates tested
☒	☐	A description of any assumptions or corrections, such as tests of normality and adjustment for multiple comparisons
☐	☒	A full description of the statistical parameters including central tendency (e.g. means) or other basic estimates (e.g. regression coefficient) AND variation (e.g. standard deviation) or associated estimates of uncertainty (e.g. confidence intervals)
☒	☐	For null hypothesis testing, the test statistic (e.g. *F*, *t*, *r*) with confidence intervals, effect sizes, degrees of freedom and *P* value noted<br>*Give P values as exact values whenever suitable.*
☒	☐	For Bayesian analysis, information on the choice of priors and Markov chain Monte Carlo settings
☒	☐	For hierarchical and complex designs, identification of the appropriate level for tests and full reporting of outcomes
☒	☐	Estimates of effect sizes (e.g. Cohen's *d*, Pearson's *r*), indicating how they were calculated

Our web collection on statistics for biologists contains articles on many of the points above.

Software and code

Policy information about availability of computer code

Data collection	The scientific software used in this study, goSPL, is available from https://github.com/Geodels/gospl and https://doi.org/10.5281/zenodo.523461. The software documentation can be found at https://gospl.readthedocs.io. SiMRiv is available at https://github.com/miguel-porto/SiMRiv. The open-source python interface for the Generic Mapping Tools (https://www.pygmt.org) is used for two-dimensional map visualization except for the ones in Supplementary Fig. 1 and 8 that were created with the open-source Paraview software (https://www.paraview.org).
Data analysis	Analysis were performed using Jupyter notebooks with the following Python libraries Numpy, scikit-image, xarray. Source codes for the generation of the figures and associated data processing have been deposited along with the raw data in the same Zenodo repository (https://doi.org/10.5281/zenodo.10889086).

For manuscripts utilizing custom algorithms or software that are central to the research but not yet described in published literature, software must be made available to editors and reviewers. We strongly encourage code deposition in a community repository (e.g. GitHub). See the Nature Portfolio guidelines for submitting code & software for further information.

Data

Policy information about availability of data

All manuscripts must include a data availability statement. This statement should provide the following information, where applicable:

- Accession codes, unique identifiers, or web links for publicly available datasets
- A description of any restrictions on data availability
- For clinical datasets or third party data, please ensure that the statement adheres to our policy

The high resolution reconstructed digital elevation model of the region is available as shape files from the Supplementary Materials in Crabtree et al. Paleo-precipitation maps from the HadCM3 coupled atmosphere-ocean-vegetation Hadley Centre climate model are available from the Bristol Research Initiative for the Dynamic Global Environment (BRIDGE) website: https://www.paleo.bristol.ac.uk/ummodel/users/Singarayer_and_Valdes_2010/new2/. Hindcasted net primary production values for Sahul produced by the LOVECLIM global circulation model are available at 1 kyr resolution from https://doi.org/10.5281/zenodo.4453767. Source codes for the generation of the figures and associated data processing have been deposited along with the raw data in the same Zenodo repository (https://doi.org/10.5281/zenodo.10889086).

Research involving human participants, their data, or biological material

Policy information about studies with human participants or human data. See also policy information about sex, gender (identity/presentation), and sexual orientation and race, ethnicity and racism.

Reporting on sex and gender	Not application to our study
Reporting on race, ethnicity, or other socially relevant groupings	Not application to our study
Population characteristics	Not application to our study
Recruitment	Not application to our study
Ethics oversight	Not application to our study

Note that full information on the approval of the study protocol must also be provided in the manuscript.

Field-specific reporting

Please select the one below that is the best fit for your research. If you are not sure, read the appropriate sections before making your selection.

☐ Life sciences ☐ Behavioural & social sciences ☒ Ecological, evolutionary & environmental sciences

For a reference copy of the document with all sections, see nature.com/documents/nr-reporting-summary-flat.pdf

Ecological, evolutionary & environmental sciences study design

All studies must disclose on these points even when the disclosure is negative.

Study description	Simulations of Homo sapiens peopling of Sahul in the Pleistocene
Research sample	The study involves existing published archaeological datasets available from Crabtree et al. (2021):<br>Crabtree, S. et al. Landscape rules predict optimal superhighways for the first peopling of Sahul. Nat. Hum. Behav. 5, DOI: 10.1038/s41562-021-01106-8 (2021). As in Crabtree et al., we removed sites younger than 35,000 years old and sites with ages more than 35,000 years old that are documented as not directly related to human occupation (e.g., ages taken on samples below archaeological deposits). We selected 35,000 years ago as a threshold to encompass ages representing the earliest phases of human occupation of the continent, and before significant societal change from the Last Glacial Maximum.
Sampling strategy	Crabtree et al. systematically compiled Australian sites published since the completion of AustArch in 2014 from the literature, ages missed in the original AustArch compilation, and extended the dataset to include New Guinea to encompass all of Sahul. We did not include archaeological sites on islands that have never formed part of the Sahul landmass (e.g., the Bismarck and Solomon Archipelagos) in the dataset.
Data collection	We did not collect any data and rely on existing published archaeological datasets available from Crabtree et al. (2021). Crabtree et al. (2021) constructed the reference archaeological site dataset for Sahul by building on the AustArch compilation; for more information on age reliability we recommend reviewing that publication.
Timing and spatial scale	We look at a period spanning from 75,000 to 35,000 years over the Sahul continent (i.e. New Guinea, mainland Australia and Tasmania) and used already published archaeological sites dated to >35,000 years old. These data are provided in our supplemental information where quality-rated ages largely following the protocol developed by Rodríguez-Rey et al. for evaluation of ages

associated with Sahul megafauna deposits. Quality rating comprised a two-step process resulting in allocation of ages to one of four categories of reliability. The first step evaluated the dating technique itself resulting in assignment of the age to one of four categories (best to worst: m*, m, B, C). The second step evaluates the strength of association of ages rated m* and m with the dating target, in this case the association of the sample with cultural deposits, resulting in a final reliability rating (best to worst: A*, A, B and C). Quality ratings for other dating techniques such as optically stimulated luminescence follow the criteria outlined in detail in Rodríguez-Rey et al. Following Crabtree et al., we reduced the dating quality of any infinite age or any age without a reported error to C. We reduced the overall quality rating of any age > 75 ka to a C, because this is beyond the accepted age of initial peopling of Sahul. We reduced the overall quality rating of any age to a C where the authors reporting the age state that it is unreliable, or where the authors note that it is not associated with cultural material.

Data exclusions	No data were excluded from the archaeological sites available in Crabtree et al. (2021)
Reproducibility	We run a series of 10,000 mechanistic simulations and opt for a probabilistic assessment of the large set of simulations to obtain statistically meaningful results. All attempts to repeat the experiment were successful.
Randomization	This is not relevant to our study.
Blinding	Blinding is not applicable to our study.

Did the study involve field work? ☐ Yes ☒ No

Reporting for specific materials, systems and methods

We require information from authors about some types of materials, experimental systems and methods used in many studies. Here, indicate whether each material, system or method listed is relevant to your study. If you are not sure if a list item applies to your research, read the appropriate section before selecting a response.

Materials & experimental systems

n/a	Involved in the study
☒	☐ Antibodies
☒	☐ Eukaryotic cell lines
☒	☐ Palaeontology and archaeology
☒	☐ Animals and other organisms
☒	☐ Clinical data
☒	☐ Dual use research of concern
☒	☐ Plants

Methods

n/a	Involved in the study
☒	☐ ChIP-seq
☒	☐ Flow cytometry
☒	☐ MRI-based neuroimaging

Plants

Seed stocks	This is not relevant to our study.
Novel plant genotypes	This is not relevant to our study.
Authentication	This is not relevant to our study.
